# Nicotinic Acetylcholine Receptor Dysfunction in Addiction and in Some Neurodegenerative and Neuropsychiatric Diseases

**DOI:** 10.3390/cells12162051

**Published:** 2023-08-11

**Authors:** Ana Sofía Vallés, Francisco J. Barrantes

**Affiliations:** 1 Bahía Blanca Institute of Biochemical Research (UNS-CONICET), Bahía Blanca 8000, Argentina; sofiavalles@gmail.com; 2 Biomedical Research Institute (BIOMED), Faculty of Medical Sciences, Pontifical Catholic University of Argentina—National Scientific and Technical Research Council, Av. Alicia Moreau de Justo 1600, Buenos Aires C1107AFF, Argentina

**Keywords:** nAChRs, health, disease, Alzheimer disease, Parkinson disease, schizophrenia spectrum disorders, epilepsy, addiction

## Abstract

The cholinergic system plays an essential role in brain development, physiology, and pathophysiology. Herein, we review how specific alterations in this system, through genetic mutations or abnormal receptor function, can lead to aberrant neural circuitry that triggers disease. The review focuses on the nicotinic acetylcholine receptor (nAChR) and its role in addiction and in neurodegenerative and neuropsychiatric diseases and epilepsy. Cholinergic dysfunction is associated with inflammatory processes mainly through the involvement of α7 nAChRs expressed in brain and in peripheral immune cells. Evidence suggests that these neuroinflammatory processes trigger and aggravate pathological states. We discuss the preclinical evidence demonstrating the therapeutic potential of nAChR ligands in Alzheimer disease, Parkinson disease, schizophrenia spectrum disorders, and in autosomal dominant sleep-related hypermotor epilepsy. PubMed and Google Scholar bibliographic databases were searched with the keywords indicated below.

## 1. Introduction

Nicotinic acetylcholine receptors (nAChRs) are members of the pentameric ligand-gated ion (cation) channel (pLGIC) superfamily, which includes neurotransmitter receptors in metazoa and other ion channels in prokaryota [1]. The various nAChR subunits (α1–α10, β1–β4, γ, ε, and δ) are encoded by 17 genes in vertebrates. nAChRs typically assemble in a combination of two pairs of αβ subunits and an accessory subunit, giving rise to a hetero-pentameric structure [2]. Homopentameric structures containing only α subunits are possible as well [2], allowing for a wide combinatorial diversity of neuronal and muscle-type nAChRs (fetal and adult) with distinctive pharmacological and biophysical properties [3,4,5,6,7,8].

nAChRs occur in multiple dynamic conformational states. In the absence of a ligand, the receptor rests with its ion channel in a closed state. In the presence of an agonist, the receptor protein rapidly shifts to an open state that allows the influx of small cations. The open channel conformer can either return to the closed state or transit to a desensitized (and closed) state. In the desensitized state, the nAChR is unable to be activated by ligand binding.

During brain development, neuronal nAChRs contribute to neurogenesis, neurite outgrowth, and synaptic maturation [9,10,11]. In the human brain, neuronal nAChRs are commonly found in the basal forebrain, hippocampus, cerebellum, and temporal cortex. These brain locations participate in learning, cognition, and memory [11]. Among neuronal nAChRs, heteromeric α4β2 nAChRs and homomeric α7 nAChRs are the most abundant subtypes, whereas combinations such as α3β4, α3β2, and α6β2β3 nAChRs are less common and are generally restricted to specific brain regions [12,13]. 

Cholinergic signaling is actively involved in the modulation of the finely tuned balance between excitatory and inhibitory neurotransmission in the brain. nAChR activation by the endogenous neurotransmitter acetylcholine (ACh) promotes the release of Ca^2+^ from intracellular stores and the induction of long-term potentiation (LTP) that favors a depolarized state of the neuron. Activation of nAChRs at pre-synaptic compartments favors the release of several neurotransmitters, including dopamine (DA), norepinephrine, γ-aminobutyric acid (GABA), and glutamate (Glu) [14]. Activation of α4β2 nAChRs allows the modulation of synaptic architecture by regulating the abundance of dendritic spines and heterologous synaptogenesis [15]. Post-synaptically located α7 nAChRs can regulate Glu receptors, thereby modulating synaptic plasticity and GABAergic interneuron activity [16,17]. Furthermore, α7 nAChRs can modulate network excitability, as either their activation or inhibition at the prelimbic cortex promotes the induction of LTP [18]. Thus, cholinergic receptors have a major role in the regulation of neural excitability and plasticity. Likewise, impaired depolarization of the postsynaptic membrane affects communication between neurons [19]. 

nAChR are expressed in non-neuronal cells as well; α7 nAChRs are present on peripheral immune cells, in astrocytes, in microglia, and in endothelial cells where they mediate neuroprotection and the inflammatory response to different insults [20,21,22].

α7 and α4β2 nAChRs have been associated with the pathogenesis of a range of neurological disorders, e.g., Alzheimer disease (AD) [23], schizophrenia spectrum disorders [24], Parkinson disease (PD) [25], nocturnal frontal lobe epilepsy [26], autism spectrum disorder [27], attention deficit hyperactivity disorder [28], and depression [29], to name some of the most relevant ones. Dysfunctional nAChRs have been implicated in non-neurological diseases as well, e.g., small-cell lung carcinomas and diabetes [30]. The predominant α7 and α4β2 nAChRs and the role of other nAChR subtypes in different diseases is currently the subject of basic and clinical research. One example is involvement of the α9 nAChR subunit in the pathophysiology of neuropathic pain [31,32] and participation of the α6 subunit in sensory processing and pain [31,33]. Muscle-type nAChR dysfunctions cover a wide clinical spectrum. Those due to inherited mutations can be associated with muscle weakness, myasthenia gravis, or congenital myasthenic syndromes [34,35]. Knowledge of nAChR involvement in neuromuscular, neurological, and psychiatric disorders makes these receptors critical targets for drug development [3,27,36,37,38,39]. 

Understanding the complex neurobiological mechanisms implicated in addiction is key to the development of a therapeutic protocol/strategy for the treatment of what is today considered a chronic neuropsychiatric disorder. Epidemiological studies have provided information on the association of certain neurological diseases, such as PD and schizophrenia, with heavy smoking habits and decreased brain expression of specific nAChRs. Therefore, information on the role of these receptors in such pathophysiological states is the first of many steps leading to therapeutic intervention.

Many if not all neurological diseases present an inflammatory component. nAChRs play a central role in the cholinergic anti-inflammatory pathway and in the regulation of immune functions in AD, PD, and schizophrenia spectrum disorders. Hence, activation of this pathway has emerged as a therapeutic tool in the amelioration of the neuroinflammatory component of these diseases. Neuroinflammatory comorbidities not only decrease the quality of life of the patient, they represent a social, emotional, and financial burden to society. Research on how nAChRs are altered in disease will undoubtedly contribute to the development of therapies to reverse or at least hamper the progression of these debilitating diseases. 

The fundamental role played by nAChRs as modulators of neurotransmitter release determines that dysfunction or mutations of the genes encoding these receptor subunits affect many cognitive functions and favor the occurrence of a wide range of pathologies, such as addiction, neuropsychiatric and neurodegenerative diseases and epilepsy, as critically discussed in this review.

## 2. Addiction

Addiction can be defined as a chronic neuropsychological disorder in the form of an intense compulsive urge to seek immediate sensory rewards; it is characterized by functional alterations in brain circuits that participate in reward, memory, and self-control, leading to maladaptive behaviors [40]. The hallmark of addiction involves what are colloquially known as the four Cs: the appearance of craving, the compulsion to “use”, the loss of control, and adverse consequences [41]. An understanding of the neurobiological mechanisms and central actors involved in the development of the different stages of addiction is fundamental. Today, it is acknowledged that several neurotransmitters and neuromodulators regulate brain reward areas acting at the level of either the ventral tegmental area (VTA) or the nucleus accumbens (NAc). These areas of the brain balance and modulate emotions, stress, and interoception. The intake of any substance that disbalances the multiple neurotransmitter-specific neuroplasticity circuitries will induce neuroadaptations with different effects on the individual [41].

**Figure 1:**
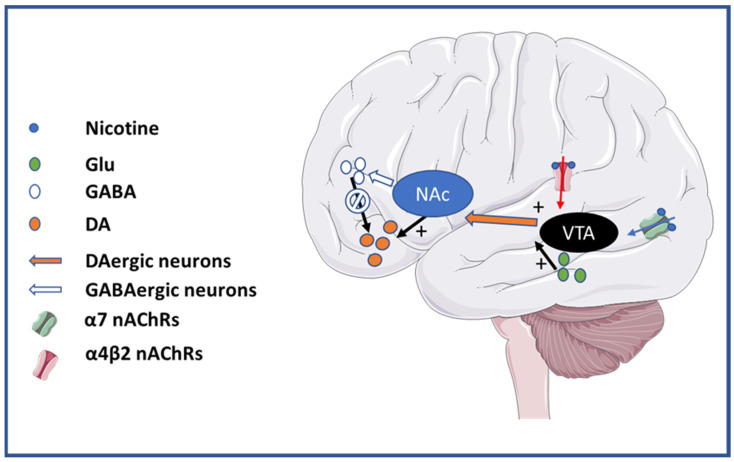
Schematic diagram showing key nuclei and pathways involved in addiction with strong participation of nAChRs. Nicotine binding to α7 and α4β2 nAChRs at the ventral tegmental area (VTA) promotes the initiation of addictive behavior by favoring the release of dopamine (DA) in the nucleus accumbens (NAc).

Nicotine (1-methyl-2-[3-pyridyl] pyrrolidine), mainly found in tobacco, is considered a highly addictive drug involving all of the criteria contained in the four Cs [42]. In its uncharged form it can permeate the plasma membrane and enter the brain, where it can change to its charged form to bind and trigger nAChRs [12,43]. By activating a variety of nAChRs located in DAergic neurons in the VTA that projects to the NAc, nicotine promotes the release of DA in the NAc, a key step in the initiation of addiction [43] (Figure 1). 

In the VTA, nicotine activates α4β2 nAChRs, promoting GABA release in GABAergic neurons and thereby inhibiting dopamine release. However, because α4β2 nAChRs rapidly desensitize upon agonist binding, the GABAergic flux to the dopaminergic (DAergic) neurons is brief. In parallel, nicotine activates α7 nAChRs in presynaptic neurons of the VTA, promoting Glu release, which can in turn enhance DAergic release [42]. Because α7 nAChRs have lower affinity for nicotine than α4β2 nAChRs, they are less prone to desensitization in the presence of the nicotine concentrations found in the brain upon smoking [44,45,46]. Thus, nicotine addiction is promoted through the combinatory action of a reduction in the inhibitory GABAergic input to DAergic neurons and the potentiation of glutamatergic afferents to dopamine-releasing neurons. Therefore, nAChRs are central actors in the modulation of DA release, and consequently in the initiation of nicotine addiction [47,48,49,50].

Unlike the natural endogenous agonist ACh, which is rapidly hydrolyzed by acetylcholinesterase, nicotine cannot be removed from the synaptic cleft [6,43,51,52]; this constant exposure to nicotine triggers several neuroadaptations. Chronic nicotine exposure leads to nAChR upregulation, with modifications in receptor assembly, trafficking, and degradation that contribute to maintaining adequate brain homeostasis [5,53,54,55]. The neuroadaptations taking place in neurotransmitter systems as a consequence of nicotine exposure are considered to participate directly in nicotine addiction [52,56,57].

Through the mesolimbic pathway, nicotine–nAChR interactions mediate reward and reinforcement effects [42]. The habenula has been found to be involved in the regulation of feelings such as fear, anxiety and depression [43,58,59,60,61]. The aversive effects of nicotine withdrawal are mediated through the medial habenula-interpeduncular (MHb-IPN) [62,63].

The principal nAChR subunits expressed in the mesolimbic pathway are the α4, α6, α7, and β2 subunits, with the α3, α5, and β4 subunits being mostly expressed in the MHb-IPN [64]. It has been hypothesized that α5 subunits comprise ∼20% of functional nAChRs in rat MHb neurons that project to IPN [65]. Knockdown of α5 nAChRs in the MHb-IPN pathway further suggests that nicotine exerts stimulatory effects on α5-containing nAChRs [66]. Nicotine and other addictive substances have been reported to interact with the α3β4 nAChR expressed in the MHb-IPN circuit [67,68], and it has been proposed that they mediate drug- or psychostimulant-seeking behavior [69]. 

In addition to the many environmental and social factors affecting nicotine addiction, individual genetic factors play an important role [42]. The risk of developing nicotine addiction has been associated with genetic variations in genes that encode for nAChRs, particularly those located in the chromosomal region 15q25 (*CHRNA5-CHRNA3-CHRNB4* gene cluster) [42], in chromosome 8 (*CHRNB3–CHRNA6* gene cluster) [70], and in *CHRNA2* [71]. The cited reports suggest a strong association between single-nucleotide polymorphism (SNPs) in nAChR genes and the number of cigarettes smoked per day, the age onset of daily smoking, and chronic smoking behaviors in adolescence and adulthood. Recent use of knockout/knock-in mice has contributed to our understanding of different behavioral phenotypes related to nicotine addiction. The rewarding DA-mediated effects along with the aversive consequences of nicotine withdrawal preclude active smokers from stopping the habit, and can often motivate relapse after periods of abstinence.

In line with studies showing nAChR involvement in cognition, inhibitory control, and decision-making mechanisms [72], nicotine activation of nAChRs has been shown to enhance attention in animal and human studies [73,74,75]. Nicotine-induced enhancement of cognition has been reported to be weaker in non-smokers than in smokers [76]. The higher incidence of smoking among individuals with psychiatric illnesses such as schizophrenia spectrum disorders may indicate that patients with these conditions smoke to ameliorate the attentional deficits associated with their disease condition [77,78]. In line with the self-medication hypothesis, it has been reported that schizophrenia patients have lower expression of nAChRs [79,80,81] and as such may smoke in order to up-regulate their nAChR and thereby augment their nicotine level [79,80,82].

Many epidemiological and longitudinal studies in recent years have revealed that most tobacco users consume cannabis as well [83,84,85]. Cannabis and nAChR receptors co-distribute in the same brain areas, suggesting that the two systems can engage in cross-talk [86]. Tobacco and cannabis are the most common drugs of abuse consumed by adolescents and young adults [87,88]. The co-use of these drugs has been suggested to produce mutually reinforcing effects and a decrease in adverse effects [89]. Several studies have indicated that consumption of Δ-9-tetrahydrocannabinol (THC), the main addictive component in *Cannabis sativa*, is associated with anxiogenic-like effects, working memory impairments, and ataxia [88,89]. These adverse THC effects appear to be reduced upon nicotine administration [90,91,92,93,94].

The *CHRNA2* gene in chromosome 8 has recently been identified as one of the risk loci for both smoking behavior and nicotine dependence [71,95]. A recent study found that individuals with cannabinoid use disorder present reduced expression of the *CHRNA2* gene in the cerebellum, suggesting that the gene that encodes for the α2 nAChR subunit may be involved in the susceptibility to developing this disorder. Furthermore, a negative correlation between the gene expressions of *CHRNA2* and *CNR1* (cannabinoid receptor 1) in the cerebellar cortex and cerebellar nuclei has been reported [87]. Participation of the homomeric α7 nAChR has been linked to the rewarding effects of cannabinoid use, while the α4β2 nAChR subtype has been associated with a reduction in cannabinoid-induced ataxia, and as such with a reduction in cannabinoid-induced motor impairment. In addition, the potential roles of the α5, α3, and β4 nAChR subunits in cannabinoid use disorder, particularly in tolerance- and withdrawal-associated symptoms, have been addressed [87].

To summarize, nicotine and/or cannabis addiction induces several neuroadaptations involving nAChRs in brain regions that modulate the mesolimbic reward system and the MHb-IPN withdrawal syndrome. Preclinical studies have provided a wealth of information on alterations to the neurocircuitry due to chronic consumption of these substances. The great diversity of nAChR subunits along with genetic differences in gene clusters that code for these subunits are important features of addiction and should be considered jointly in the design of therapeutic approaches.

## 3. Central and Peripheral Inflammation

The cholinergic system is involved in the modulation of inflammation in the central and peripheral nervous systems [11,31,96]. Cholinergic receptors are expressed in neurons, glial cells (microglia, astrocytes), and immune cells (e.g., macrophages) [11,31,96]. In the nervous system, neuroinflammation is a necessary process to restore the altered homeostasis caused by infections, trauma, and neurodegenerative diseases [97]. 

In the CNS, neuroinflammation comprises a dynamic multistage physiological response orchestrated by microglia and astrocytes [97]. Both types of cells are needed to support and sustain adequate neuronal function. In most neurodegenerative diseases a chronic inflammatory state is present. Under these circumstances, microglia remain activated for prolonged periods, with detrimental consequences for neuronal cells [97]. Activated microglial cells secrete various inflammatory molecules that may lead to neuronal dysfunction and degeneration [98]. Likewise, during sustained inflammation astrocytes release pro-inflammatory cytokines and prostaglandins that can alter neuronal function and the blood–brain barrier [99].

**Figure 2:**
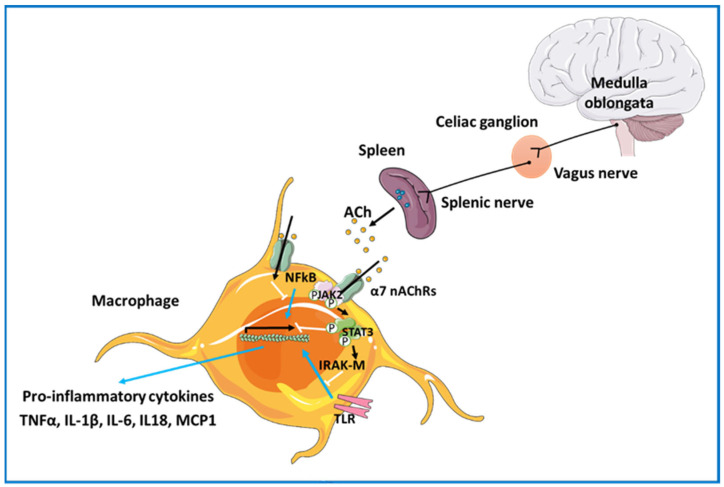
Schematic diagram of nodes and tracks of the cholinergic anti-inflammatory pathway. The connection between the vagus and the splenic nerve via the celiac ganglion promotes noradrenaline release (blue circles) and activation of splenic T-cells. T-cells release acetylcholine (ACh) that can bind to α7 nAChR on immune cells such as macrophages, inhibiting the release of pro-inflammatory cytokines. Activation of α7 nAChR inhibits the nuclear factor kappa-light-chain-enhancer of activated B cell (NFkB) translocation to the cell nucleus and activation of a Janus kinase 2 (JAK2)–signal transducer and activator of transcription 3 (STAT3)-mediated signaling pathway. In parallel, activation of α7 nAChRs may up-regulate the expression of interleukin-1 receptor-associated kinase M (IRAK-M), which can negatively regulate innate Toll-like receptor (TLR)-mediated immune responses, contributing to cholinergic anti-inflammatory effects.

Both microglia and astroglia express α7 nAChRs, and several studies have demonstrated that this nAChR subtype exerts neuroprotective effects in the brain [20,100,101,102,103]. As such, glial α7 nAChRs are considered potential therapeutic targets in neurodegenerative diseases [104]; furthermore, α7 nAChR agonists have been reported to provide neuroprotection against various toxic insults including β-amyloid [105], MPTP (in vivo) and MPP+- or LPS (in vitro) [106]. The ionotropic activity of α7 nAChRs is neuron-specific. In non-neuronal cells, metabotropic activity is prevalent downstream of α7 nAChR activation [97]. Activation of α7 nAChR expressed in glial cells promotes the activation of phospholipase C (PLC), in turn inducing the enhanced production of inositol trisphosphate (IP3) [97]. This second messenger can bind to its receptor, located in the endoplasmic reticulum, and induce the release of Ca^2+^, while the cation mediates a decrease in phosphorylation, causing the activation of kinases involved in neuroinflammation. α7 nAChR activation in glial cells regulates the synthesis and release of inflammatory molecules such as TNFα, IL-6, and nitric oxide [98,107,108]. In addition, α7 nAChRs expressed in astrocytes have been shown to mediate anti-inflammatory effects by inhibiting the nuclear factor kappa-light-chain-enhancer of the activated B cell (NFkB) pathway and activation of the nuclear factor erythroid 2-related factor 2 (Nrf2) pathway [104]. Hence, α7 nAChR in glial cells appears to play a significant role in the modulation of neuroinflammation in the CNS (Figure 2). 

Peripheral immune cells additionally express nAChRs [109], which can be activated by the endogenous neurotransmitter. Several studies have shown that specific ligands of the α7, α9, and α10-containing nAChRs can modulate the release of inflammatory cytokines from peripheral immune cells [109,110,111,112]. Although the precise mechanisms of signal transduction in these cells have not been fully elucidated, it is known that when ACh binds to α7 nAChRs on cytokine-producing cells, such as macrophages, activation of a signaling cascade via the Janus kinase 2 (JAK2) signal transducer and activator of transcription 3 (STAT3) takes place [100,113,114]. As a result, STAT3 translocates to the cell nucleus and interferes with the binding of NFkB to the DNA (Figure 2). The latter event prevents the transcription of genes that encode for inflammatory cytokines such as interleukin 1β, 6, and 8, TNF-α, or monocyte chemoattractant protein-1 (MCP1) (Figure 2). 

An alternative anti-inflammatory mechanism initiated through the JAK2-STAT3 pathway [109,111,112,115] has been described. Activation of this pathway would promote the expression of interleukin-1 receptor-associated kinase M (IRAK-M), which can negatively regulate the innate Toll-like receptor (TLR)-mediated immune responses. The TLRs comprise a family of receptors that are necessary for the initiation of innate immune responses [115]. Thus, activation of α7 nAChRs in peripheral immune cells would suppress the production of pro-inflammatory molecules by inhibiting downstream inflammatory signals resulting from TLR activation [109].

nAChRs containing the α9/α10 subunits have been suggested to partake in these anti-inflammatory mechanisms [109]. Activation of α9/α10-containing nAChRs in human monocytes and whole blood cultures inhibit release of IL-1β, TNF-α, and IL-6 [116,117,118]. The mechanism by which these receptors relay this inflammatory protection appears to be the same as that mediated by α7 nAChR through activation of the JAK2/STAT3 pathway [109]. Indeed, the cholinergic anti-inflammatory pathway links the nervous system with the immune system to counteract inflammatory activation [110,119]. The neural circuit by which the vagus nerve interacts with the peripheral immune system to provide anti-inflammatory action involves activation of the splenic nerve. When splenic nerve fibers are activated, they release noradrenaline. Splenic T-cells release ACh in response to noradrenaline binding to β2 adrenergic receptors. In turn, released ACh can activate α7 nAChR in peripheral immune cells (Figure 2) and confer anti-inflammatory protection.

Recently, Simon and coworkers further described that in addition to the already known α7-mediated anti-inflammatory effects via vagus nerve stimulation, splenic nerve terminals that release noradrenaline can interact directly with noradrenergic receptors in splenic myeloid cells and exert anti-inflammatory effects [120]. Vagus nerve stimulation therapy was introduced in the 1980s to treat epilepsy [121]. The procedure is a non-invasive tool that has been applied more recently to AD [122], PD [123], and schizophrenic [124] patients. Furthermore, preclinical studies performed in rodents have shown that vagus nerve stimulation limits the accumulation of β-amyloid plaques, while clinical studies have shown promising results in the modulation of cognition.

In summary, neuroinflammation constitutes an ubiquitous pathology in the various CNS diseases discussed in this review. As the best characterized nicotinic receptor subtype in the immune system [96], stimulation of α7 nAChR is emerging as a promising target to counteract neuroinflammatory processes and a major contributor to the restoration of CNS homeostasis. A better understanding of the functionality of nAChR in both central and peripheral immune cells and their ability to abrogate inflammatory processes is of great clinical relevance. 

## 4. Alzheimer Disease

AD is considered the most common form of dementia among elderly persons [125]. The development of amyloid senile plaques, containing amyloid peptides, and deposits of neurofibrillary tangles rich in tau protein are pathognomonic postmortem hallmarks of AD [126]. Reports contend that these plaques and deposits occur in the brain long before the clinical manifestations of AD become evident [125,127]. Moreover, it is these pathological alterations that have been suggested to induce neuronal dysfunction associated with clinical dementia, a strong decline in memory and cognitive functions, and a deterioration in the visual and motor coordination manifested in symptomatic AD dementia [128].

Cortical nAChRs are markedly reduced in the brains of AD patients, explaining the cholinergic deficits associated with AD [129]. In particular, altered expression levels and function of α7 nAChR have been described in AD [110,125]. Reduced α7 nAChR levels have been shown to correlate with β-amyloid (Aβ) plaque deposition and cognitive impairment [130,131,132], and there is strong evidence that α7 nAChR interacts directly with the Aβ peptide [125]. The Aβ peptide can bind to α7 nAChR at the surface of neurons with very high (pM) affinity [133]. Endocytic internalization of the α7 nAChR–Aβ complex and the ensuing Aβ aggregation then promotes the phosphorylation of microtubular tau protein, leading to the formation of neurofibrillar tangles [133].

In recent years, innate immune activation has been ascribed an important role in both the pathogenesis and progression of AD. While microglia can interact with Aβ and Aβ precursor protein (APP) through membrane receptors and clear Aβ from the brain through phagocytosis, it can release pro-inflammatory cytokines that result in damage to the surrounding neurons [134]. Therapeutic interventions targeting microglia in neurodegenerative diseases are currently in their infancy [134]. Future therapeutic approaches targeting microglial activation in AD should aim at specifically inhibiting the release of inflammatory factors without interfering with microglia’s beneficial effects on Aβ clearance.

Several genes encoding for immune receptors have been linked to AD development [135]. It is now recognized from preclinical and clinical evidence that systemic inflammation can affect the brain in many ways and can lead to the development of neurodegenerative diseases including AD [135]. Indeed, preclinical data have provided a large body of evidence on the association between peripheral inflammation and AD pathology [135]. Likewise, several clinical reports have described how systemic inflammation caused by specific environmental factors is associated with an increase in cognitive decline in AD [136,137,138]. Activation of α7 nAChRs expressed in rat hippocampal astrocytes was able to counteract this inflammatory scenario by reducing the Aβ protein load [139]. However, Aβ concentration changes as AD progresses; hence, α7 nAChRs may play different roles as the disease develops. At low picomolar concentrations, Aβ triggers the conversion of α7 nAChR to a desensitized conformation that is nevertheless able to respond to agonists and exert anti-inflammatory action, whereas at high nanomolar concentrations Aβ acts as a negative modulator of the receptor and possesses associated neurotoxicity [140,141,142]. Thus, the concentration of Aβ should be critically evaluated in terms of the benefits of α7 nAChR stimulation therapies. Furthermore, because of the strong affinity interaction between Aβ and α7 nAChR, the pharmacological selection of a competitive Aβ antagonist has been challenging [143]. Different drug candidates have emerged, including partial and allosteric modulators of the α7 nAChR. Many trials have been abandoned, however, either because of poor efficacy or high toxicity [143]. There are significant gaps to be filled between preclinical and clinical data in the interests of better AD therapeutic strategies.

The *CHRFAM7A* gene is exclusively found in humans [142,144,145,146]. This gene is the product of the partial duplication of exons 5 to 10 of the α7 nAChR-encoding gene *CHRNA7* [147,148]. The *CHRFAM7A* human-specific gene that lacks the N-terminal domain of the CHRNA7 subunit codifies the dupα7 protein [142]. Thus, the agonist binding domain is absent in the dupα7 protein. The dupα7 protein cannot by itself assemble into functional nicotinic receptors. However, in combination with at least two α7 nAChR subunits it can form functional ion channels [142], albeit exerting a dominant negative effect on the latter [149]. Furthermore, polymorphism as well as a two base pair deletion of the *CHRFAM7A* on exon 6 has been described [147]. These genetic modifications translate into the dupΔα7 protein [147]. The number of *CHRFAM7A* copies varies [142], some individuals being non-carriers of the *CHRNA7* duplication and others expressing one or two copies. The occurrence of the Δ2bp allele varies between different ethnic groups [142]. There is evidence that the number of *CHRFAM7A* copies carried by an individual may affect the response of α7 nAChR-positive allosteric modulators (PAM), agonists, and antagonists [142,150,151,152]. Interestingly, the expression of dupα7 has been associated with a protective role during the accumulating phase of Aβ [150]. The authors cited above describe the presence of *CHRFAM7A* as mitigating Aβ uptake in cells; thus, its expression could exert a protective role when Aβ concentrations are above physiological levels [150]. Previous reports using a transgenic mouse model of AD [153] on a knockout α7 nAChR mouse [154] showed that deletion of the α7 nAChR gene ameliorated cognitive deficiency and further improved synaptic physiology [155]. Altogether, these studies highlight the importance of α7 nAChR in the pathophysiology of the cognitive impairment associated with AD [133]. *CHRFAM7A* is expressed in non-neuronal cells as well; therefore, it can alter the anti-inflammatory effects mediated via α7 nAChR activation [149,156]. In one study, LPS-induced inflammatory responses were reported to down-regulate *CHRFAM7A* expression at the mRNA and protein levels. Conversely, CHRNA7 mRNA was upregulated [156].

Approximately 25% of the AD population are non-carriers of the *CHRFAM7A* gene. Considering that preclinical drug testing is carried out in animal models, any molecule screened to target the α7 nAChR will benefit a quarter of the population at most [150,151,152]. Therefore, future preclinical models examining the *CHRFAM7A* gene in greater detail should turn the focus to the rest of the population [151]. Non-carrier individuals should experience better outcomes based on preclinical data of AD drug trials, while more specific research is needed to understand the impact of *CHRFAM7A* on the pathogenesis of AD.

## 5. Parkinson Disease

**Figure 3:**
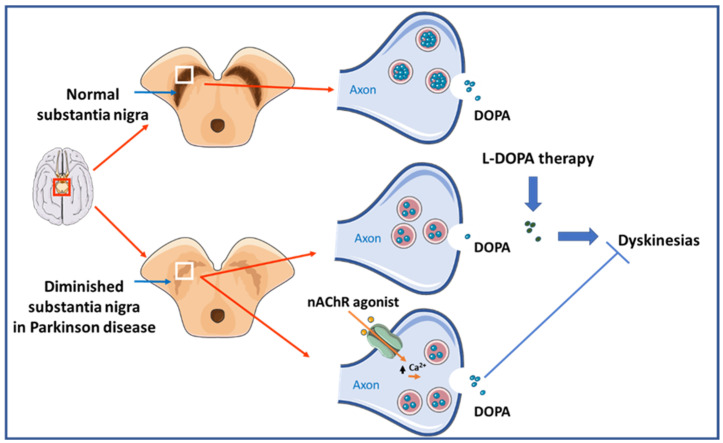
Dopaminergic neurons from the substantia nigra are substantially reduced in PD. Current treatments are aimed at counterbalancing the reduction of dopamine (DOPA) by exogenous administration of L-DOPA. nAChR agonists may offer amelioration of dyskinetic symptoms in PD by promoting the endogenous release of DOPA.

The second most frequent neurodegenerative disease is PD. PD patients exhibit motor deficits, cognitive decline, and sleep and affective disturbances, and they progressively lose DAergic neurons from the substantia nigra (Figure 3).

The most common treatment for PD is replacement therapy with L-DOPA to enhance DA transmission, with beneficial effects on PD-associated motor dysfunction. However, this approach does not suffice to improve other PD-associated symptoms, nor does it help to prevent the progression of the disease. In addition, a common side effect of L-DOPA therapy is the induction of dyskinesias that are incapacitating.

Activation of nAChRs at presynaptic terminals can enhance DA release from DAergic neurons. Promising results for treating PD have been obtained in animal models and human studies involving activation of the nigrostriatal pathway by nAChR agonists [157,158,159,160]. The potential cognitive-enhancing properties of nAChR-targeting drugs may be of additional benefit to those patients suffering cognitive decline [25,159,161].

PD neuroprotection from toxin-induced DAergic cell loss by nAChR agonist administration has been described in animal models [162]. Indeed, the specific activation of α7 nAChRs in several PD animal models has proven beneficial for ameliorating PD-associated symptoms and for its antidyskinetic effects. In contrast, administration of methyllycaconitine, a specific α7 nAChR antagonist, hinders neuroprotection [106,162]. 

Activation of nAChRs containing the β2 subunit, as is the case with the abundant α4β2 subtype, has shown protection against 6-OHDA-induced nigrostriatal damage in rodents [25]. The nAChR β2 subunit has been suggested to mediate this neuroprotective effect, as nigrostriatal damage was not prevented in α4 subunit-knockout mice models lacking α4β2 nAChRs [163]. The β2 subunit has been found to modulate the expression of induced dyskinesia in several nonhuman primate models [164,165,166,167].

Nicotine-mediated α7 nAChR activation has been shown to produce an inhibitory effect on L-DOPA-induced dyskinetic side effects in nonhuman primate models [168,169]. However, α7 nAChR seems not to be the specific mediator of this effect, as mutant mice lacking the α7 subunit reduce the L-DOPA-induced abnormal movements when exposed to nicotine. 

Neuroinflammatory processes are present in PD, and increased density of astrocytes and active microglia is observed as well. Microglial cells initiate the immune response, and astrocytes surround the area so as to localize the secretion of pro-inflammatory cytokines [170,171]. In consequence, when activated, microglia and astrocytes release pro-inflammatory cytokines in PD, and degeneration of dopaminergic neurons can occur [172]. Recent preclinical research has focused on preventing microglia activation to delay the progression of the disease [172,173], although the use of these drugs in clinical practice is far from being a reality. PD patients accumulate α-synuclein in the form of Lewy bodies [174]. This α-synuclein accumulation in PD contributes to neuroinflammation by promoting the release of pro-inflammatory molecules from glial cells, which has neurotoxic effects [174]. Although the origin of α-synuclein aggregation remains uncertain, two hypotheses have been proposed [175]. In the first hypothesis, α-synuclein accumulation is purported to arise in the brain and project into the peripheral autonomic nervous system. A second hypothesis postulates that α-synuclein pathology originates in the gastrointestinal tract and reaches the brain via the vagus nerve [175]. Because α7-nAChR is expressed in glial cells and in peripheral immune cells, this receptor subtype is envisaged as a possible therapeutic target to reduce neuroinflammation in PD. The expression of α7 nAChR in astrocytes is considered a novel therapeutic strategy for the treatment of PD [104]. Likewise, vagus nerve stimulation is becoming more accepted as a non-invasive therapeutic method to tackle neuroinflammation in PD [123].

## 6. Schizophrenia Spectrum Disorders

Schizophrenia and associated disorders have a profound negative impact on the quality of life of patients. These complex chronic neuropsychiatric disorders have an early onset [176]. The symptoms experienced by schizophrenic patients have been classified as positive symptoms (delusions, hallucinations), negative symptoms (social withdrawal, anhedonia), and cognitive deficits (learning and memory deficits, alogia) [177]. Altered neurotransmission has been proposed as the basic common pathophysiological mechanism in schizophrenia [178]. Augmented levels of pro-inflammatory cytokines have been found in schizophrenic patients as well [179].

Several forms of schizophrenia spectrum disorders have a heritable genetic component. However, no single causative gene has been reported to date. Trubetskoy and coworkers [180] recently studied the genomes of 76,755 individuals with schizophrenia as well as 243,649 healthy (control) participants. Their study demonstrated the occurrence of 342 common genetic variants that could increase the risk of developing schizophrenic disorders. Therefore, as with other neurological and neuropsychiatric diseases, it is likely that a combination of many genes and environmental factors contribute to the pathogenesis of this group of disorders.

Regarding alterations in cholinergic neurotransmission in schizophrenic patients, studies performed in postmortem hippocampus, cortex, thalamus, and striatum of schizophrenic patients have revealed that expression of α7 nAChR is lower than in control patients [24,181]. The lower number of receptors in schizophrenic patients does not appear to be region-specific. 

The gene encoding for α7 nAChR is located in chromosome 15q14, which is linked to genetic transmission of schizophrenia spectrum disorders [177,182]. As is the case with AD, carriers of the *CHRFAM7A* gene mutation are associated with certain forms of this spectrum [142,177,183,184]. Additionally, the reduced expression of α7 nAChRs may result from increased expression and insertion of heteromeric dupα7/α7 nAChRs [177,185,186]. 

An endophenotype of schizophrenia spectrum disorders is the presence of P50 auditorily evoked response deficits. In these patients, the involvement of a nicotinic receptor is clearly apparent [187], as administration of nicotine transiently normalizes the P50 deficit. Furthermore, a strong link between α7 nAChRs and sensory gating deficits has been reported in some patients with schizophrenia [142,188,189]. Therefore, it is not surprising that these patients use smoking as a form of self-medication (see the above section on Addiction). Indeed, about 80% of patients with schizophrenia consume tobacco products, a figure that dramatically contrasts with 25% among the general population [190,191]. These statistics are underscored by the observation that therapeutic strategies targeting α7 nAChR show beneficial effects [142,192,193,194,195].

The presence of heteromeric dupα7/α7 nAChRs, increased *CHRFAM7A*, and/or reduced *CHRNA7* expression in the prefrontal cortex has been reported in patients with bipolar disorder and schizophrenia spectrum disorders [142,196,197]. However, there are contradictory findings in the literature regarding the association of these variants with some forms of schizophrenia, probably due to differences in the ethnic groups and number of subjects under study and the phenotype under consideration [183,196,198]. Future preclinical trials should include these genetic variants in order to gain greater insight into the possible involvement of nAChR genes as risk factors in the development of schizophrenia spectrum disorders in order to improve therapeutic outcomes.

## 7. Epilepsy

Epilepsy comprises many syndromes characterized by the chronic occurrence of seizures [199]. The latter results from excessive neuronal activity in the brain [200]. Epileptic syndromes show heterogeneous origins, mechanisms, and clinical manifestations [201]. Because gliosis and microgliosis have been described in epilepsy, it is evident that this malady shares a neuroinflammatory component with other neuronal diseases [202]. Therefore, future preclinical and clinical studies should consider including agents to reduce neuroinflammation in their therapeutic approaches. 

A high proportion of individuals presenting alterations in neurodevelopment suffer comorbid seizures. Again, nAChRs have been shown to play an important role in regulation of the excitatory microcircuitry that leads to seizures, and a vast body of evidence implicates nAChR dysregulation in epileptiform activity [199].

Both nAChRs and muscarinic receptors (mAChRs) have been associated with epilepsy, as their hyperstimulation can lead to the onset of seizures [203,204]. Different nAChR subtype mutations have been linked with genetic sleep-related epilepsy [26,199,205,206,207,208]. nAChRs can induce epileptogenic effects both during development and in adult stages due to their participation in synaptogenesis and the regulation of mature synaptic circuit excitability. Hyperactivation of M1 mAChRs upon application of the muscarinic agonist pilocarpine has been used as a model of temporal lobe epilepsy, to induce transient status epilepticus, and to generate chronic epileptic seizures [203,204,209].

In vivo studies have shown that nicotine doses over 2–3 mg/kg in rodents suffice to induce tonic–clonic convulsions [210,211]. Clinical data suggest that seizures can occur after multiple applications of transdermal nicotine patches [212]. In addition, repeated subconvulsive doses of nicotine in mice have been used as kindling agents [213]. Interestingly, it has been suggested that the sex dependency of nicotine-induced kindling is related to the lower availability of antioxidant defenses in females [214]. nAChR antagonists prevent the induction of pro-convulsive activity [215,216,217]. More difficult to explain is the fact that high concentrations of nAChR antagonists can have pro-convulsive activity as well [215,218]. Reports indicate that mutant mice deficient in α5 and/or β4 nAChR subunits are less prone to developing seizures [219,220]. One proposed mechanism of nicotine kindling and activity-dependent nAChR-induced seizures argues that the two pathologies are due to the induction of glutamatergic overactivation [221]. Autosomal dominant nocturnal frontal lobe epilepsy (ADNFLE), now renamed (AD)SHE, or (Autosomal dominant) sleep-related hypermotor epilepsy [222], was first linked to a missense mutation on *CHRNA4* (α4S248F) [26,206,223]. More recently, *CHRNA2* was found to be linked to ADSHE [224]. In these forms of epilepsy, hypermotor seizures or tonic–dystonic postures that last on average ∼30 s are observed. The new terminology, ADSHE, reinforces the concept that sleep-related seizures are not exclusively nocturnal and enhances the importance of hypermotor seizures as the central feature of the pathology. In addition to motor hyperactivity, ADSHE families that carry the CHRNA4 and CHRNB2 mutations present cognitive disabilities, mental retardation, and schizophrenia-like symptoms [199]. How nAChR-expressing mutant subunits are linked to the onset of the pathologies of ADSHE requires knowledge of the roles these receptors play in multiple neuronal circuits during development and in adulthood. 

Deletion of *CHRNA7* in mice does not alter the induction of seizures by nicotine administration [225,226]. Furthermore, human-based studies have revealed a genetic predisposition to developing an idiopathic form of generalized epilepsy [227,228] and to some of the neurodevelopmental disorders accompanied by seizures [227,229,230] when microdeletions of the chromosome region 15q13.3 that codes for *CHRNA7* are present. These microdeletions have been associated with phenotypes that lead to schizophrenia- and epilepsy-related alterations in animal studies [231]. Additional studies are required in order to understand the possible implications of α7 nAChRs in epilepsy. Deeper knowledge of the molecular mechanisms by which nAChR mutations induce ADSHE in response to agonist/antagonist exposure is essential for the formulation of pharmacological strategies targeting these forms of epilepsy.

## 8. Concluding Remarks

By regulating neuronal excitability, immunity, inflammation, neuroprotection, and the release of other neurotransmitters or targeting the receptors for other neurotransmitters, nAChRs modulate multiple physiological, behavioral, and pathophysiological processes. Different levels of expression of nAChRs in specific brain regions and at different neurodevelopmental stages can be affected by dysfunction and lead to disease. The design of specific nAChR ligands, including PAMs, able to target specific diseases and be tailored to subtle variations in each pathophysiological scenario, calls for several translational gaps to be filled in preclinical and clinical trials. The inter-individual variability in genes that encode for nAChR subunits needs to be carefully considered in future personalized therapies, along with adequate genetic screening. In addition, nAChR-based or nAChR-targeted therapeutic strategies must include multiple genetic variants in order to improve the potential of these drugs in all populations.
